# Prevalence and Antimicrobial Resistance of *Campylobacter coli* and *Campylobacter jejuni* Isolated from Pig Guts, Pig Feces, and Surface Swabs from the Cutting Tables at Slaughterhouse and Taverns in Southern Benin

**DOI:** 10.1155/2022/5120678

**Published:** 2022-09-29

**Authors:** Alidehou Jerrold Agbankpe, Sylvain D. Kougblenou, Tamegnon Victorien Dougnon, Alida Oussou, Elodie Gbotche, Charles Hornel Koudokpon, Brice Boris Legba, Lamine Baba-Moussa, Honore Sourou Bankole

**Affiliations:** ^1^Research Unit in Applied Microbiology and Pharmacology of Natural Substances, Research Laboratory in Applied Biology, Polytechnic School of Abomey-Calavi, University of Abomey-Calavi, P.O. Box 2009, Cotonou 01, Benin; ^2^Laboratory of Food Microbiology, Ministry of Health, P.O. Box 418, Cotonou 01, Benin; ^3^Laboratory of Biology and Molecular Typing in Microbiology, Faculty of Science and Technology, University of Abomey-Calavi, P.O. Box 1604, Cotonou 05, Benin

## Abstract

*Campylobacter* food-borne infections are a serious public health problem. In Benin, there is a proliferation of pork consumption in several forms. This study aims to determine the prevalence and the antimicrobial resistance of *Campylobacter coli and Campylobacter jejuni* strains isolated from pig guts, pig feces, and surface swabs from the cutting tables in southern Benin. For this purpose, 200 samples of pig guts, 40 samples of swabs from the cutting table surface, and 8 samples of pig feces were collected and subjected to bacteriological examination. The method used for the identification of bacteria was microbiological culture combined with molecular identification by PCR. The identified strains were then subjected to antibiotic susceptibility testing according to the methodology recommended by the EUCAST. Antibiotic profiles were compared between strains isolated from pig guts, pig feces, and cutting table surfaces on the one hand and among the different sampling sites on the other hand. The results obtained show that 47.6% of the samples analyzed were contaminated by *Campylobacter* spp. Molecular identification revealed 34.7% of *Campylobacter coli* and 9.3% of *Campylobacter jejuni*. The study of antimicrobial susceptibility showed resistance to ciprofloxacin, 44% to ampicillin, 23.9% to erythromycin, 11% to gentamicin, and 10.1% to amoxicillin + clavulanic acid. In total, 90.8% of the isolated *Campylobacter* strains were multidrug resistant. The use of antimicrobials in livestock production systems has increased considerably, which could explain, at least partially, the prevalence of *Campylobacter* and the resistance of strains to antibiotics. To limit the risk of *Campylobacter* food-borne infections, it is therefore important to include *Campylobacter* in the list of pathogens to be tested during sanitary quality control of meat and meat products in Benin.

## 1. Introduction

Human *Campylobacter*iosis is primarily caused by *Campylobacter jejuni* (*C. jejuni*) and *Campylobacter coli* (*C. coli*) [1]. Since 2005, *Campylobacter*iosis has been the main food-borne gastrointestinal infection reported in humans in the European Union [2]. Over the past decade, incidence and prevalence of *Campylobacter*iosis have increased worldwide, with approximately 500 million cases of gastroenteritis reported each year [3]. *Campylobacter* transmission mainly occurs following exposure to farm animals with such infections, with subsequent transmission through the food chain to retail food products [4, 5]. Risk factors for infection include consumption of undercooked meat, unpasteurized dairy products or contaminated drinking water, and direct contact with farm animals [6]. Farm animals such as poultry and pig are common hosts and important reservoirs of *Campylobacter*. *C. jejuni* is considered prevalent in poultry [7], while pigs are mostly implicated as reservoirs of *C. coli* [8]. However, *Campylobacter jejuni* also hosts pigs, but at a lower rate [9]. Meat obtained from poultry is the most common source of *Campylobacter* bacteria, but pork also represents source of infection with these microorganisms [10]. The pigs are often subclinically infected with *Campylobacter* spp., and contamination of meat during processing remains a significant risk to food security [11].

The majority of human *Campylobacter* infections cause self-limiting gastroenteritis and do not require specific treatment. However, severe, prolonged, or systemic infections in the immunocompromised and vulnerable population and children may require antimicrobial therapy [12]. The resistance to antimicrobials of foodborne pathogenic bacteria is an important concern for human health [13]. This is due to the fact that many antimicrobials used to treat human infections are used in animal husbandry as prophylactics and feed supplements, which has been shown by the selection of resistant isolates that can affect human health if they get into food chains [14]. Antimicrobial-resistant *Campylobacter* infections have been associated with increased mortality and morbidity [15].

The Food and Agriculture Organization of the United Nations (FAO) estimated that the national herd of pigs in Benin was a total of 504,000 head in 2018 [16]. There are no official statistics on the number of pig farms in Benin. However, there is a strong demand for pork in Benin [17]. Thus, there is a proliferation of restaurants selling pork in various forms. However, the genus *Campylobacter* is not included in the list of microorganisms searched for in the framework of the sanitary qualification of meat in Benin. And yet, it is a bacterium that is the leading cause of food-borne illness in the world today [18]. This study aims to determine the prevalence and the antimicrobial resistance of *Campylobacter coli* and *Campylobacter jejuni* strains isolated from pig guts, pig feces, and surface swabs from the cutting tables in southern Benin.

## 2. Materials and Methods

### 2.1. Study Area

**Figure 1:**
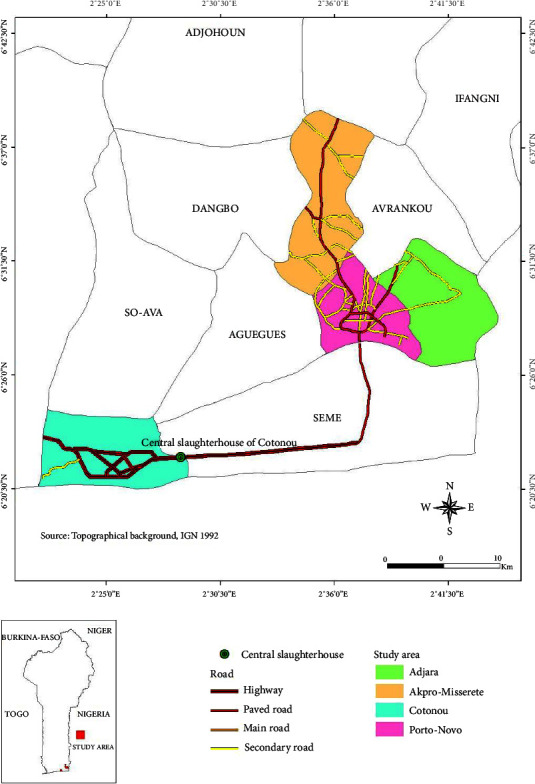
Map of southern Benin showing the study area and the central Cotonou slaughterhouse.

This study was conducted in southern Benin, mainly at the central *slaughterhouse* of Cotonou and in a few taverns in the municipalities of Cotonou, Adjarra, Akpro-Missérété, and Porto-Novo where pork is served on the menu (Figure 1). The Cotonou Central Slaughterhouse was chosen for the health checks carried out there on the animals that are to be slaughtered and especially the fact that most restaurants in Cotonou buy meat from this slaughterhouse. This is therefore meat from a controlled environment. It is only here that the samples of pig feces were taken; it is certain that at this location, there is more of a chance of finding a steady supply of the animals. The choice of municipalities was motivated by the fact that they are part of the provinces of southern Benin where pork is most produced and consumed [19].

## 3. Methods

### 3.1. Sampling

#### 3.1.1. Sample Size

The minimum sample size (*n*) was estimated from the Schwartz formula: *n* = (z^2^·*p*·*q*)/*d*^2^, where *p* = prevalence; *p* = 0.20 (because the prevalence in Benin is 20%) [20]; *q* = 1 − *p* = probability that a sample is not contaminated with *Campylobacter*; *z* = accuracy level according to the normal distribution law (for a 95% confidence level, *z* = 1.96); and *d* = tolerated margin of error for this study equal to 0.05. Thus, *n* = ((1.96)2 × 0.22 × 0.8)/(0.05)^2^; *n* = 245.86 or *n* = 246 samples. The sample size used for this study was 248 samples.

#### 3.1.2. Allocation of Samples and Sampling Technique

The taverns from which the samples were taken were randomly selected, but the owners' consent was obtained before sampling. Similarly, the consent of the head of the central slaughterhouse in Cotonou was obtained prior to the collection of the various samples. At the Cotonou central slaughterhouse, 5 pig guts sampling points, 1 pig feces sampling point, and the large cutting table have been identified for sampling. Thus, 8 pig guts samples were taken from each sampling point (8 × 5 pig guts samples), 8 pig feces samples, and 8 cutting tables swab samples were taken, respectively. According to their attendance and according to the number of pigs (1 to 2) slaughtered per day, eight (08) taverns were identified in each of the four (04) municipalities selected. In each tavern selected, 5 samples of pig guts and 1 sample of cutting table swabs were taken, i.e., 48 samples per municipality. Thus, 200 pig guts samples, 40 swab samples from the cutting tables surfaces, and 8 pig feces samples were collected (Table 1).

Using a pair of sterile scissors and forceps, approximately 250 g of pig guts were collected in sterile collection bags. Pig feces (approximately 250 g) were collected in sterile collection bags using sterile spatulas. A surface area of 100 cm^2^ of each of the cutting tables was sampled after cleaning and before any handling of pig carcasses. A stainless-steel template of 10 cm side, designed for this purpose, was used. After sampling, the swabs used were each introduced into a tube containing 10 mL of Preston broth (CM 0067 Oxoid Ltd. Basingstoke, UK) enriched with fresh sheep blood and Preston supplement (CM 0183Oxoid Ltd. Basingstoke, UK). All the samples thus taken were labeled and transported to the laboratory in a cooler containing cold packs, apart from the samples of pig feces, which were transported in bags. The microbiological analyses were carried out within 4 hours after the samples were taken.

### 3.2. Microbiological Analysis

#### 3.2.1. Sample Enrichment, Isolation, and Purification of *Campylobacter* spp

The pork and feces samples were analyzed according to the modified NF EN ISO 10272-1 standard, described by Bankolé et al. [20] and taken up by Kougblenou et al. [21]. As for the cutting table surface swab samples, they were directly incubated in a microaerophilic atmosphere at 42°C for 24 hours, these samples then underwent the same microbiological analysis procedure as the other samples. Thus, 45 mL and 225 mL of Preston broth (CM 0067 Oxoid Ltd. Basingstoke, UK) enriched with fresh sheep blood and Preston supplement (CM 0183 Oxoid Ltd. Basingstoke, UK) were, respectively, added to 5 mL of brain-heart broth (BK026HA Biokar Diagnostics, Rue Delizy, France) preculture (swab enriched) and 25 g of pig guts or pig feces sample contained in a sterile stomacher bag. After grinding and mixing with a stomacher, the bag was then hermetically sealed and then incubated at 42°C in a microaerophilic atmosphere for 48 hours.

After 48 h, the subcultures were streaked on the Preston *Campylobacter* (PC) (CM 0689 Oxoid Ltd. Basingstoke, UK) and Karmali *Campylobacter* (KC) (REF 610200 Liofilchem Srl, Via Scozia, Italy) agar plates, and the dishes were incubated in a microaerophilic atmosphere at 42°C. for 48 h. A characteristic colony of *Campylobacter* was then taken, respectively, from PC and KC agars and inoculated onto nutrient agar (NG) (BK046HA Biokar Diagnostics, Rue Delizy, France) enriched with fresh sheep blood. These plates were incubated in a microaerophilic atmosphere at 37°C for 36 h. The pure cultures obtained were stored in MH broth (BK048HA Biokar Diagnostics, Rue Delizy, France) with glycerol (30%) at −37°C for further analyses.

#### 3.2.2. Phenotypic Identification of *Campylobacter* spp Strains

The identification of the strains of *Campylobacter* spp was carried out according to the methodology described by Kougblenou et al. [21]. After Gram staining, the biochemical tests performed such as catalase, oxidase, hydrolysis of hippurate, production of nitrate reductase, fermentation of sugars, production of hydrogen sulfide, and gas were carried out. In addition, growth at 25 and 42°C, and antibiotyping were performed. *Campylobacter jejuni* ATCC 29428 and *Campylobacter coli* ATCC 33559 were the *Campylobacter* reference strains used. *Pseudomonas aeruginosa* ATCC 27853, *Staphylococcus aureus* ATCC 29213, and *Escherichia coli* ATCC 25922 were used to validate the tests and techniques used.

#### 3.2.3. Identification by PCR of Isolated *Campylobacter* Strains

*DNA Extraction*. The different samples subcultured in nutrient agar were ground in 200 *μ*l of 2% CTAB. After 5 minutes in a water bath at 65°C, the ground material was mixed with 200 *μ*l of chloroform then centrifuged at 14,000 revolutions per minute for 5 minutes. The supernatant was gently collected in another tube with 200 *μ*l of isopropanol and then centrifuged at 12000 rpm for 15 minutes. The pellet was preserved with 200 *μ*l of 70% ethanol. The whole was centrifuged at 14000 rpm for 5 minutes. The contents of the tube were gently inverted in order to preserve the pellet, which was then dried for a moment on the bench. Finally, 20 *μ*l of distilled water was added to the pellet left in suspension on the bench overnight or half a day.

*Polymerase Chain Reaction (PCR)*. The search for *Campylobacter* in the samples were carried out by PCR using in the first place specific primers for *Campylobacter* all species (16SrRNA (816 bp) were the targeted gene): C412F 5′-GGATGACACTTTTCGGAGC-3′ and C1228R 5′-CATTGTAGCACGTGTGTC-3′ [22, 23]. Then the identification of isolates at the rank of species *C. jejuni* and *C. coli* was carried out using specific primers. The nucleotide sequences of these primers are: *C. jejuni* (mepA (413 bp) was the target gene): CJmapAN3F 5′-TGGTGGTTTTGAAGCAAAGA-3′ and CJmapAN3R 5′-GCTTGGTGCGGATTGTAAA-3′ [22, 24]; *C. coli* (ceuE (330 bp) was the target gene) CCceuEN3F 5′-AAGCGTTGCAAAACTTTATGG-3′ and CCceuEN3R 5′-CCTTGTGCGCGTTCTTATT-3′ [22, 24].

The PCR reaction was performed in a final volume of 25 *µ*l containing 1x amplification buffer, 1.5 mM MgCl2, 0.2 mM of each deoxynucleotide Trisphosphate (dNTP), 0.1 mg/ml bovine serum albumin (BSA), 10pmoles of each primer, 1 U of taq, and 1 *µ*l of extracted DNA. The amplification program is an initial denaturation at 95°C for 5 minutes followed by 35 cycles of denaturation at 94°C for 40 seconds, hybridization at 50°C for 40 seconds, elongation at 72°C for 40 seconds, and finally a final elongation at 72°C for 7 minutes. The amplicon was run in a 2% agarose gel mixed with ethidium bromide. For each PCR reaction, two positive controls were performed using the reference strains *Campylobacter jejuni* ATCC 29428 and *Campylobacter coli* ATCC 33559.

Determination of the antimicrobial resistance profile of *Campylobacter* isolates, determination of *Campylobacter* antimicrobial susceptibility has only been performed on strains isolated and identified by PCR, using the disc diffusion method (CA-SFM, [25]. The antimicrobials tested were such as: ampicillin (AMP) (10 *µ*g), gentamicin (10 *µ*g) (GM), erythromycin (15 *µ*g) (E), ciprofloxacin (CIP) (5 *µ*g), tetracycline (30 *µ*g) (TE), and amoxicillin + clavulanic acid (20 *µ*g) (AMC). These antimicrobials were chosen according to EUCAST recommendations (2018). From a pure culture of 18–24 h of incubation, a bacterial suspension with an opacity of 0.5 McFarland was prepared and diluted 1 : 10. After inoculation by swab on Mueller–Hinton agar containing 5% fresh sheep blood (63902 Bio-Rad Laboratories, Inc, Marnes-La-Coquette, France), and application of antibiotic discs (BioMérieux SA, Craponne, France), the plates were incubated in a microaerophilic atmosphere for 24 h at 37°C.

### 3.3. Statistical Analysis

The data were analyzed with the statistical software *R* version 3.6.1. Chi-square and Fisher's exact tests were performed to compare the results of tested samples and antimicrobial susceptibility testing. The Kappa concordance coefficient was calculated to assess the concordance of the phenotypic identification with the PCR identification of the isolated *Campylobacter* strains. The difference was significant when *p* < 0.05. Additionally, 95% confidence interval was also determined for antibiotic resistance rates.

## 4. Results

### 4.1. Results of Bacteriological Culture of Samples

Of the 248 samples analyzed, 47.6% (*n* = 118) were positive for *Campylobacter* culture. Feces samples were the most contaminated with *Campylobacter* at 75%. They were followed by those from cutting tables (57.5%) and pig guts (44.5%) (Table 2). This difference in contamination rate between samples is not statistically significant (*p* > 0.05). Similarly, there was no significant difference in the contamination rates of the samples according to their origin (*p* > 0.05).

### 4.2. Phenotypic and Molecular Identification of Isolated *Campylobacter* Species

**Figure 2:**
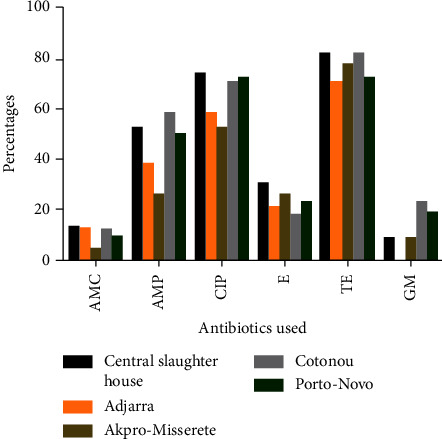
Distribution of the resistance of *Campylobacter* strains identified according to the sampling locations. AMC: amoxicillin + clavulanic acid; AMP: ampicillin; CIP: ciprofloxacin; E: erythromycin; TE: tetracycline; GM: gentamicin.

Phenotypic identification revealed 89 strains of *C. coli* and 21 strains of *C. jejuni*. Molecular identification of these strains showed that the 21 phenotypically identified *C. jejuni* strains were confirmed by PCR, i.e., a 100% confirmation rate. As for the *C. coli* strains, 86 of the 89 strains identified were confirmed by PCR, i.e., a confirmation rate of 96.6%. Thus, of the remaining three *C. coli* strains not confirmed by PCR, two were identified as *C. jejuni,* and one as *Campylobacter* spp. (Table 3) (Figure 2). In summary, we were able to identify and confirm 86 strains of *C. coli* and 23 strains of *C. jejuni* with the Kappa concordance coefficient equal to 0.796 (*p* ≤ 0.001) (Table 3).

### 4.3. Distribution of *C. coli* and *C. jejuni* Species according to Samples and Sampling Locations

*Campylobacter coli* strains were more representative (39.6%) in the commune of Adjarra, with a strong presence in samples of pig guts (31.3%). As for *Campylobacter jejuni* strains, the cutting table samples from the communes of Adjarra, Akpro-Missérété, and Porto-Novo were the most contaminated by this strain with a percentage of 8.3% (Table 4).

### 4.4. Antibiotic Resistance of *Campylobacter* Strains Identified

The antimicrobial susceptibility testing was performed on *Campylobacter* strains identified by PCR. The *C. coli* strains isolated from pig guts, cutting tables, and pig feces showed a high resistance to tetracycline in the proportions of 86.7%, 66.7%, and 66.7%, respectively. As for the *C. jejuni* strains, they showed strong resistance to amoxicillin and gentamicin (100%), especially for the cutting table samples. All the strains isolated (100%) from pig feces samples showed resistance to tetracycline, with 66.7% and 33.3% of the strains, respectively. *C. coli* and *C. jejuni* were isolated. As for strains isolated from fecal samples, 50% were resistant to erythromycin, with 66.7% and 33.3% of *C. coli* strains, respectively. This difference between the percentages of resistance to erythromycin of the strains isolated from the different types of samples is statistically significant (*p* = 0.0248) (Table 5).

### 4.5. Antibiotic Resistance of *Campylobacter* Strains according to Sampling Locations

According to the distribution of resistance, *C. coli* and *C. jejuni* strains showed various resistance patterns. The strongest resistance was observed in *C. coli* and *C. jejuni* isolates from all municipalities, against ciprofloxacin, tetracycline, and ampicillin. There is also resistance to erythromycin in the strains isolated from the samples from the slaughter and the town of Akpro-Missérété (Figure 2).

### 4.6. Profiles of Identified Multidrug-Resistant *Campylobacter* Strains

All isolated *Campylobacter* strains were resistant to at least one antimicrobial. In total, 22.9% of the strains were resistant to a single antimicrobial and 37.6% to two antimicrobials. According to the different types of samples, 57.7% of the strains isolated from pig guts samples were multidrug resistant, especially to AMP-CIP-TE (20%) and CIP-E-TE (10.0%) combinations. As for the different *Campylobacter* species identified, 47% of *C. coli* strains and 23% of *C. jejuni* strains showed resistance to three antimicrobials (Table 6).

## 5. Discussion

Bacteria of the genus *Campylobacter* are listed as important food pathogens. This study aims to determine the prevalence and the antimicrobial resistance of *Campylobacter coli* and *Campylobacter jejuni* strains isolated from pig guts, pig feces, and surface swabs from the cutting tables in southern Benin. Thus, through our results, we found the prevalence of *C. coli* and *C. jejuni* in the pork production chain in southern Benin is 34.7% and 9.3%, respectively (Table 3). A study conducted on 80 hog farms in Ontario to determine the prevalence of *Campylobacter* showed the presence of *C. coli* and *C. jejuni* in pork production with prevalence of 92% and 0.2%, respectively [26]. Similarly, on 139 samples of pork carcasses in Chitwan, Nepal, there is a prevalence of 76% of *C. coli* and 24% of *C. jejuni* [27]. These results show that in the pork production chain (from breeding to slaughter), there is a high circulation of *C. coli* than *C. jejuni* in terms of *Campylobacter* infections. This observation is supported by the study of Tîrziu et al. [28] where the *Campylobacter* species identified in raw chicken meat were *C. coli* (70%) and *C. jejuni* (30%) [28]. However, our results are contrary to the observations of Carrique-Mas et al. [29], who reported the predominance of *C. jejuni* and named it as the main *Campylobacter* species to colonize pigs. This same observation has been made in several studies where the *C. jejuni* species was predominant over that of *C. coli* in poultry meat [30, 31].

Additionally, strains of *Campylobacter* spp were found more in pig guts than in feces and surface swabs from cutting tables. These results show that the contamination of pig guts is not cross-contamination, but that the animals were contaminated from the breeding farms. This situation raises the problem of the emergence of strains of *Campylobacter* in pig farms in Benin. Already in 2019, the work of Kouglenou et al. had shown the presence of *Campylobacter* strains in chicken meat produced in Benin [31]. Thus, the circulation of pathogenic strains of *Campylobacter* in the livestock sector in Benin is no longer an allegation but a reality. The work of Mdegela et al. showed that the caecum was a favorite area for the multiplication of *Campylobacter* [32]. In addition, thanks to its minimal oxygen concentration, the caecum is a favorable habitat that promotes the survival of *Campylobacter* species. Thus, there is a risk of *Campylobacter* food-borne infection among consumers of pig guts in Benin. The low proportion of contaminated cutting table surface shows there is insufficient cleaning and noncompliance with hygiene rules in taverns in Benin.

The results of this study showed that there are as many samples contaminated by *Campylobacter* in the taverns of the municipalities considered. The municipalities in which this study was carried out are located in the south of Benin and have practically the same demographic characteristics apart from Cotonou, which is the economic capital of Benin with a strong demography. But the common point between these municipalities is that pork is heavily consumed there with a large number of taverns serving this meat. Thus, the presence of *Campylobacter* strains in pig guts and on the surfaces of cutting tables does not depend on one municipality to another.

Several studies have shown the multiresistance of *Campylobacter* isolates to antimicrobials. The same is true in our study where 57.7% of the *Campylobacter* strains isolated were multidrug resistant. This multidrug resistance of strains could be explained by the inconsistent or sporadic application of antimicrobial management measures in animal production systems [33]. Moreover, in recent years, the use of antimicrobials in animal husbandry systems has increased dramatically, which could explain, at least in part, the prevalence of *Campylobacter* and the emergence of antimicrobial resistance [34–36]. In our study, 77.1% of strains are resistant to tetracycline, 65.1% to ciprofloxacin, 44% to ampicillin, and 23.9% to erythromycin. In addition, 81% of *C. coli* strains and 19% of *C. jejuni* strains showed resistance to tetracycline. In South Africa, Uaboi-Egbenni et al. [37] showed that 50–100% of *Campylobacter* strains isolated from two pig farms were resistant to erythromycin [37]. Several studies have shown the resistance of *Campylobacter* strains to ciprofloxacin, tetracycline, and erythromycin [28, 30, 38–40]. Thus, the results of this study justify the emergence of multidrug-resistance *Campylobacter* strain in the pork production chain in Benin. This study should serve as a baseline for policy decisions with a view to monitoring multidrug-resistant *Campylobacter* strains in Benin, given that fluoroquinolone-resistant *Campylobacter* is on the list of priority pathogens of the World Health Organization.

## 6. Conclusions

This study shows the presence of *Campylobacter* strains in the pork production chain in southern Benin. Pig guts are the most contaminated samples. Thus, pork carcasses could be contaminated by cross-contamination during handling and cutting. There is therefore a high risk of *Campylobacter* food-borne infections among consumers of pork in Benin. It is therefore urgent that the search for *Campylobacter* on samples of pork and other meat products be integrated into the routine microbiological control of meat products in Benin. The strains of *Campylobacter* isolated were mostly multidrug resistant. This poses the problem of the emergence of multidrug-resistant strains of *Campylobacter* in livestock in Benin. Thus, it is important that measures should be taken in the livestock sector in Benin to reduce this emergence. In addition, this study being limited, we did not search for the different resistance genes in order to present the mapping of circulating genes in the pork production chain in Benin. This study deserves to be deepened to present the complete situation of Benin in terms of *Campylobacter* infection (animal and human health).

## Figures and Tables

**Table 1:** Distribution of samples according to their nature and origin.

Origin of the samples	Type of samples	Number of site(s) sampled	Number of samples taken per site	Sample size taken	Total
Central slaughterhouse	Pig guts	5	8	40	56
	ETD	1	8	8	
	Feces	1	8	8	

Adjarra	Pig guts	8	5	40	48
	ETD	8	1	8	

Akpro-Missérété	Pig guts	8	5	40	48
	ETD	8	1	8	

Cotonou	Pig guts	8	5	40	48
	ETD	8	1	8	

Porto-Novo	Pig guts	8	5	40	48
	ETD	8	1	8	
Total					248

ETD: cutting table surface swabbing.

**Table 2:** Contamination rate of samples according to sampling locations.

Sampling locations	Sample contamination results; *n* (%)								Total sample size
	Pig guts; *n* = 40/sampling location		Cutting table swabbing; *n* = 8/sampling location		Feces; *n* = 8		Total		
	−	+	−	+	−	+	−	+	
Cotonou central slaughterhouse	25 (62.5)	15 (37.5)	4 (50.0)	4 (50.0)	2 (25.0)	6 (75.0)	31 (55.4)	25 (44.6)	56
Adjarra	21 (52.5)	19 (47.5)	2 (25.0)	6 (75.0)	—	—	23 (48.0)	25 (52.0)	48
Akpro-Missérété	20 (50.0)	20 (50.0)	3 (37.5)	5 (62.5)	—	—	23 (48.0)	25 (52.0)	48
Cotonou	24 (60.0)	16 (40.0)	5 (62.5)	3 (37.5)	—	—	29 (60.4)	19 (39.6)	48
Porto-Novo	21 (52.5)	19 (47.5)	3 (37.5)	5 (62.5)	—	—	24 (50.0)	24 (50.0)	48
Total	111 (55.5)	89 (44.5)	17 (42.5)	23 (57.5)	2 (25.0)	6 (75.0)	130 (52.4)	118 (47.6)	248

(−): culture-negative specimens; (+): culture-positive specimens; *n*: effective.

**Table 3:** Phenotypic and molecular identification of isolated *Campylobacter* species.

Samples	*n*	Identification of isolated *Campylobacter* species; *n* (%)			
		Phenotypic identification		Molecular identification	
		*C. coli*	*C. jejuni*	*C. coli*	*C. jejuni*
Pig guts	200	67 (33.5)	14 (7.0)	66 (33.0)	14 (7.0)
Cutting table swabs	40	17 (42.5)	6 (15.0)	16 (40.0)	7 (17.5)
Feces	08	5 (62.5)	1 (12.5)	4 (50.0)	2 (25.0)
Total	248	89 (35.9)	21 (8.5)	86 (34.7)	23 (9.3)

**Table 4:** Distribution of *C. coli* and *C. jejuni* according to samples and sampling locations.

Sampling locations	*n*	Samples, n (%)						Total, *n* (%)	
		Pig guts		Cutting table swabs		Feces					
		*C. coli*	*C. jejuni*	*C. coli*	*C. jejuni*	*C. coli*	*C. jejuni*	*C. coli*	*C. jejuni*
Central slaughterhouse	56	11 (19.6)	2 (3.6)	2 (3.6)	2 (3.6)	4 (7.1)	2 (3.6)	17 (30.4)	6 (10.7)
Adjarra	48	15 (31.3)	3 (6.3)	4 (8.3)	2 (4.2)	—	—	19 (39.6)	5 (10.4)
Akpro-Missérété	48	14 (29.2)	4 (8.3)	4 (8.3)	1 (2.1)	—	—	18 (37.5)	5 (10.4)
Cotonou	48	12 (25.0)	2 (4.2)	2 (4.2)	1 (2.1)	—	—	14 (29.2)	3 (6.3)
Porto-Novo	48	14 (29.2)	3 (6.3)	4 (8.3)	1 (2.1)	—	—	18 (37.5)	4 (8.3)

**Table 5:** Percentages of resistance of *C. coli* and *C. jejuni* strains to antibiotics according to samples.

Classes of antibiotics	Antibiotics used	Number of strains identified, *n* = 109 (%)	Samples								
			Pig guts, *n* = 80 (%)			Cutting table swabs, *n* = 23 (%)			Feces, *n* = 6 (%)		
			*C. coli*	*C. jejuni*	Total	*C. coli*	*C. jejuni*	Total	*C. coli*	*C. jejuni*	Total
*β*-Lactamase	AMC	11 (10.1)	6 (75.0)	2 (25.0)	8 (10.0)	—	1 (100.0)	1 (4.3)	1 (50.0)	1 (50.0)	2
	AMP	48 (44.0)	27 (75.0)	9 (25.0)	36 (45.0)	5 (55.6)	4 (44.4)	9 (39.1)	2 (66.7)	1 (33.3)	3

Fluoroquinolones	CIP	71 (65.1)	44 (83.0)	9 (17.0)	53 (66.3)	9 (64.3)	5 (35.7)	14 (60.9)	3 (75.0)	1 (25.0)	4

Macrolides	E^^*∗∗*^^	26 (23.9)	8 (57.1)	6 (42.9)	14 (17.5)	3 (33.3)	6 (66.7)	9 (39.1)	2 (66.7)	1 (33.3)	3

Tétracycline	TE	84 (77.1)	52 (86.7)	8 (13.3)	60 (75.0)	12 (66.7)	6 (33.3)	18 (78.3)	4 (66.7)	2 (33.3)	6

Aminoglycosides	GM	12 (11.0)	6 (75.0)	2 (25.0)	8 (10.0)	—	2 (100.0)	2 (8.7)	1 (50.0)	1 (50.0)	2

AMC: amoxicillin + clavulanic acid; AMP: ampicillin; CIP: ciprofloxacin; E: erythromycin; TE: tetracycline; GM: gentamicin; ^^*∗∗*^^significant difference between the percentages of erythromycin resistance of strains isolated from the different types of samples (*p* = 0.00012; *p* = 0.0248).

**Table 6:** Profiles of multidrug-resistant strains identified according to samples and *Campylobacter* species identified.

Number of antibiotics from different classes	Resistance profiles	All strains identified, *n* = 109 (%)	Samples			*Campylobacter* species identified	
			Pig guts, *n* = 80 (%)	Cutting table swabbing, *n* = 23 (%)	Feces, *n* = 6 (%)	*C. coli*, *n* = 86 (%)	*C. jejuni*, *n* = 23 (%)
3	AMP-CIP-TE	22 (20.2)	16 (20.0)	4 (17.4)	2 (33.3)	18 (20.9)	4 (17.4)
	AMP-E-TE	9 (8.3)	5 (6.3)	2 (8.7)	2 (33.3)	5 (5.8)	4 (17.4)
	AMP-CIP-E	9 (8.3)	5 (6.3)	3 (13.0)	1 (16.7)	5 (5.8)	4 (17.4)
	CIP-TE-GM	8 (7.3)	5 (6.3)	1 (4.3)	2 (33.3)	5 (5.8)	3 (13.1)
	AMC-CIP-TE	3 (2.8)	2 (2.5)	—	1 (16.7)	3 (3.5)	—
	CIP-E-TE	13 (11.9)	8 (10.0)	4 (17.4)	1 (16.7)	7 (8.1)	6 (26.1)
	AMP-CIP-GM	6 (5.5)	5 (6.3)	—	1 (16.7)	4 (4.7)	2 (8.7)
	AMP-TE-GM	6 (5.5)	5 (6.3)	—	1 (16.7)	5 (5.8)	1 (4.3)
	AMC-AMP-CIP	3 (2.8)	3 (3.7)	—	—	3 (3.5)	—
	AMC-AMP-E	2 (1.8)	1 (1.3)	—	1 (16.7)	—	2 (8.7)

4	AMP-CIP-E-TE	7 (6.4)	4 (5.0)	2 (8.7)	1 (16.7)	5 (5.8)	2 (8.7)
	AMC-AMP-E-TE	1 (0.9)	—	—	1 (16.7)	—	1 (4.3)
	AMP-CIP-TE-GM	5 (4.6)	4 (5.0)	—	1 (16.7)	4 (4.7)	1 (4.3)
	AMC-E-TE-GM	1 (0.9)	—	1 (4.3)	—	—	1 (4.3)
	CIP-E-TE-GM	2 (1.8)	1 (1.3)	1 (4.3)	—	1 (1.2)	1 (4.3)

5	AMC-AMP-CIP-TE-GM	1 (0.9)	1 (1.3)	—	—	1 (1.2)	—
	AMP-CIP-E-TE-GM	1 (0.9)	1 (1.3)	—	—	1 (1.2)	—

Total		99 (90.8)	66 (60.6)	18 (78.3)	15	67 (77.9)	32

AMC: amoxicillin + clavulanic acid; AMP: ampicillin; CIP: ciprofloxacin; E: erythromycin; TE: tetracycline; GM: gentamicin.

## Data Availability

The data used to support the findings of this study are included within the article.
